# Development of a Colloidal Gold Immunochromatographic Assay for Duck Enteritis Virus Detection Using Monoclonal Antibodies

**DOI:** 10.3390/pathogens10030365

**Published:** 2021-03-18

**Authors:** Fengli Liu, Yanxin Cao, Maokai Yan, Mengxu Sun, Qingshui Zhang, Jun Wang, Guanghua Fu, Rongchang Liu, Yu Huang, Jingliang Su

**Affiliations:** 1 Key Laboratory of Animal Epidemiology of the Ministry of Agriculture, College of Veterinary Medicine, China Agricultural University, Beijing 100193, China; liufengli0216@163.com (F.L.); laura12390@163.com (Y.C.); yanmaokai1993@126.com (M.Y.); 15101065508@163.com (M.S.); freedream@cau.edu.cn (Q.Z.); 2 Agricultural and Environmental Branch, Jiaxing Vocational and Technical College, Jiaxing 314036, China; junwang@jxvtc.edu.cn; 3 Institute of Animal Husbandry and Veterinary Medicine, Fujian Academy of Agricultural Sciences, Fuzhou 350013, China; fuyuan163@163.com (G.F.); liurongc@foxmail.com (R.L.); huangyu-815@163.com (Y.H.)

**Keywords:** duck enteritis virus, monoclonal antibody, colloidal gold immunochromatographic assay strip

## Abstract

Duck viral enteritis is a highly contagious and fatal disease of commercial waterfowl flocks. The disease occurs sporadically or epizootically in mainland China due to insufficient vaccinations. Early and rapid diagnosis is important for preventive intervention and the control of epizootic events in clinical settings. In this study, we generated two monoclonal antibodies (MAbs) that specifically recognized the duck enteritis virus (DEV) envelope glycoprotein B and tegument protein UL47, respectively. Using these MAbs, a colloidal gold-based immunochromatographic assay (ICA) was developed for the efficient detection of DEV antigens within 15 min. Our results showed that the detection limit of the developed ICA strip was 2.52 × 10^3^ TCID_50_/mL for the virus infected cell culture suspension with no cross-reactivity with other pathogenic viruses commonly encountered in commercially raised waterfowl. Using samples from experimentally infected ducks, we demonstrated that the ICA detected the virus in cloacal swab samples on day three post-infection, demonstrating an 80% concordance with the PCR. For tissue homogenates from ducks succumbing to infection, the detection sensitivity was 100%. The efficient and specific detection by this ICA test provides a valuable, convenient, easy to use and rapid diagnostic tool for DVE under both laboratory and field conditions.

## 1. Introduction

Duck enteritis virus (DEV), also named Anatid herpesvirus-1, belongs to the genus *Mardivirus*, subfamily *Alphaherpesvirinae*, of the family *Herpesviridae*. It is the causative agent for duck viral enteritis (DVE), also called duck plague. Similar to most alphaherpesviruses, the DEV virion has four structural components that include the lipid envelope, tegument, capsid and a linear double-stranded DNA genome, which is approximately 158 kb and contains 78 open reading frames predicted to encode potential functional proteins [1,2]. Birds of the family Anatidae such as ducks, geese and swans are highly susceptible to DEV infection, which can be transmitted by direct contact with DEV infected birds or indirectly by contact with a virus-contaminated environment. In China, commercial duck and goose farming is widely distributed geographically with semi-intensive and open water-based farming systems. DVE occurs sporadically or epizootically in a few waterfowl-producing areas because of insufficient vaccinations [3,4,5]. A high mortality and condemnations have caused significant economic losses. Thus, early and rapid diagnosis is a key preventive intervention that would reduce the spread of DEV and may ultimately control an epidemic. Despite the gross lesions characterized by hemorrhagic and necrotic degenerative changes in the digestive tract, lymphoid and parenchymatous organs that may be observed at necropsy, confirmation of the disease mainly relies on laboratory diagnosis methods including the identification of the agent by virus isolation or PCR detection [6,7] or serological tests based on virus neutralization in a cell culture [8,9]. These methods are time-consuming and labor-intensive, requiring qualified personnel and appropriate biosafety facilities.

Immunochromatographic assays (ICA) have been successfully used for the rapid detection of analytes in a variety of biological samples especially specific antigens and antibodies of many diseases [10]. In comparison with other laboratory-based diagnostic platform analyses, ICA has the advantages of being rapid, low-cost and easy to use. These characteristics render the assay ideally suited for on-site testing even by untrained personnel with the potential to become a common diagnostic tool not only in human health but also in veterinary medicine, food safety and environmental research [11,12,13]. Currently, many ICAs have been reported in the detection of viral infections in animals [14,15,16]. To develop a sensitive and rapid diagnostic test to detect DEV as soon as possible following the onset of disease signs, we produced monoclonal antibodies that could specifically recognize DEV antigens. We then developed a monoclonal antibody-based ICA for a DEV infection. The performance of this ICA was evaluated using samples from experimentally infected ducks.

## 2. Results

### 2.1. Production of Monoclonal Antibodies Against DEV

**Figure 1:**
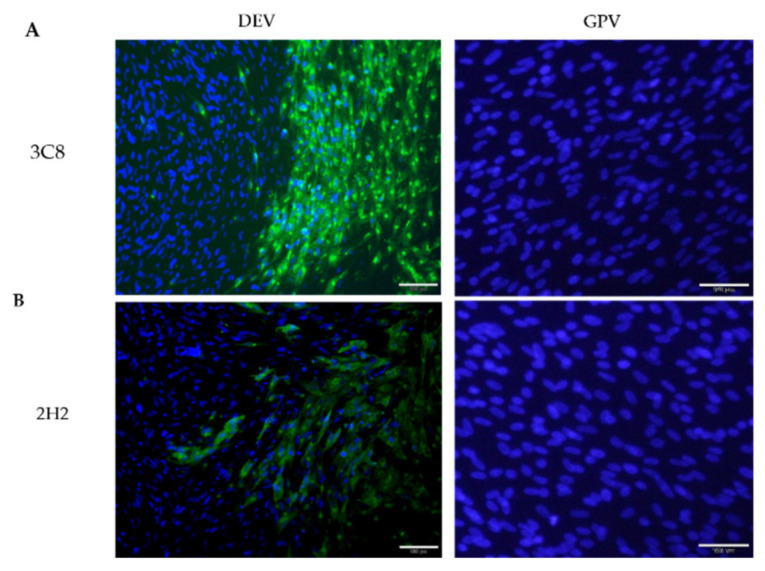
Indirect immunofluorescence staining of duck enteritis virus (DEV) and goose parvovirus (GPV) infected duck embryo fibroblasts with monoclonal antibodies 3C8 (**A**) and 2H2 (**B**) visualized with Dylight®488 (green signal). The blue signal represents nuclei counterstained with DAPI.

**Figure 2:**
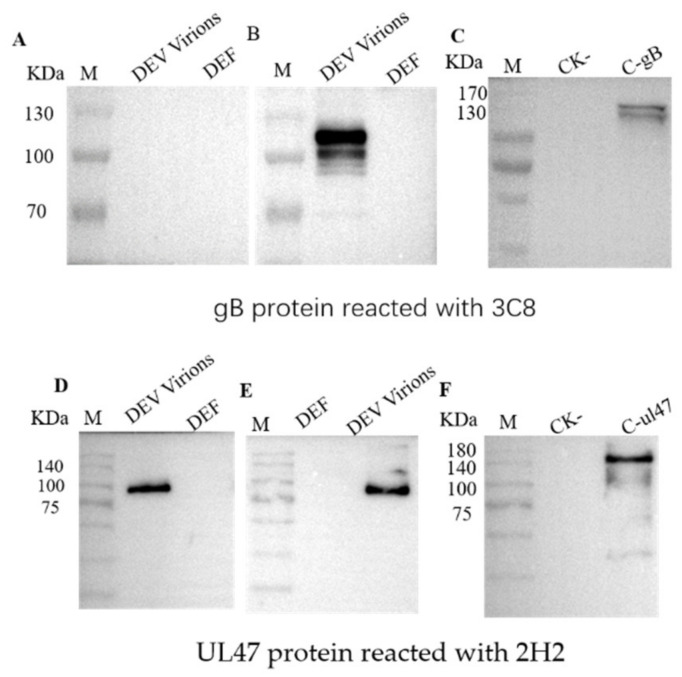
Western blot analysis of duck enteritis virus (DEV) proteins with monoclonal antibody (MAb) 3C8 and 2H2. The DEV virion proteins were reacted with the MAb 3C8 under reducing (**A**) and non-reducing (**B**) conditions; the baculovirus-expressed gB protein (**C**) was reacted with the MAb 3C8 under non-reducing conditions; the DEV virions (**D**) were reacted with the MAb 2H2 under reducing (**D**) and non-reducing (**E**) conditions; recombination UL47 proteins (**F**) were reacted with the MAb 2H2 under non-reducing conditions. DEF, duck embryo fibroblasts; C-gB, baculovirus-expressed gB protein; C-UL47, recombinant UL47 protein; CK-, negative control.

To acquire an antibody specific for DEV structural proteins, we used ultracentrifuged concentrated virions as an antigen for mouse immunization. After cloning by limiting dilution and subsequent re-screening, two stable hybridomas secreting antibodies against DEV, designated 3C8 and 2H2, were obtained and subsequently used to produce ascites fluid. Both monoclonal antibodies (MAbs) reacted strongly with the virus antigen in DEV infected duck embryo fibroblasts (DEFs) by immunofluorescence assay (IFA) detection (Figure 1). Western blotting showed that the two MAbs recognized distinct polypeptide bands of the DEV (Figure 2). It was noted that MAb 3C8 detected the target polypeptide only under non-reducing conditions of sodium dodecyl sulphate–polyacrylamide gel electrophoresis (SDS-PAGE) (Figure 2A), suggesting its epitope binding was conformation-dependent. 

To further identify the MAb-directed polypeptides, the corresponding polypeptide bands separated by SDS-PAGE were “in-gel” cut and analyzed by mass spectrometry analysis. The N-terminal amino acid sequencing results pointed to the DEV envelope protein gB and tegument protein UL47, respectively. Based on this result, genes encoding the gB and UL47 protein were amplified by PCR from the genome of the DEV strain SD and expressed in baculovirus and *E. coli BL21(DE3)*, respectively. As expected, western blots probed with the MAb 3C8 detected two bands under non-reducing conditions, corresponding with a full-length gB ( approximately 115 kDa) and one cleaved form (approximately 105 kDa) (Figure 2B). The difference in the molecular weight between the native (approximately 115 kDa) and recombinant gB (approximately 130 kDa) was due to the fusion protein fragment from the vector carrying the C-terminal bacmid. The MAb 2H2 reacted with the virion protein under reducing (Figure 2D) and non-reducing conditions (Figure 2E) and the MAb also recognized the *E. coli*-expressed recombinant DEV UL47 (about 140 KDa) (Figure 2F). These results indicated that the selected MAbs targeted the DEV envelope protein gB and tegument protein UL47.

### 2.2. ICA Assembly, Sensitivity and Specificity Determination

**Figure 3:**
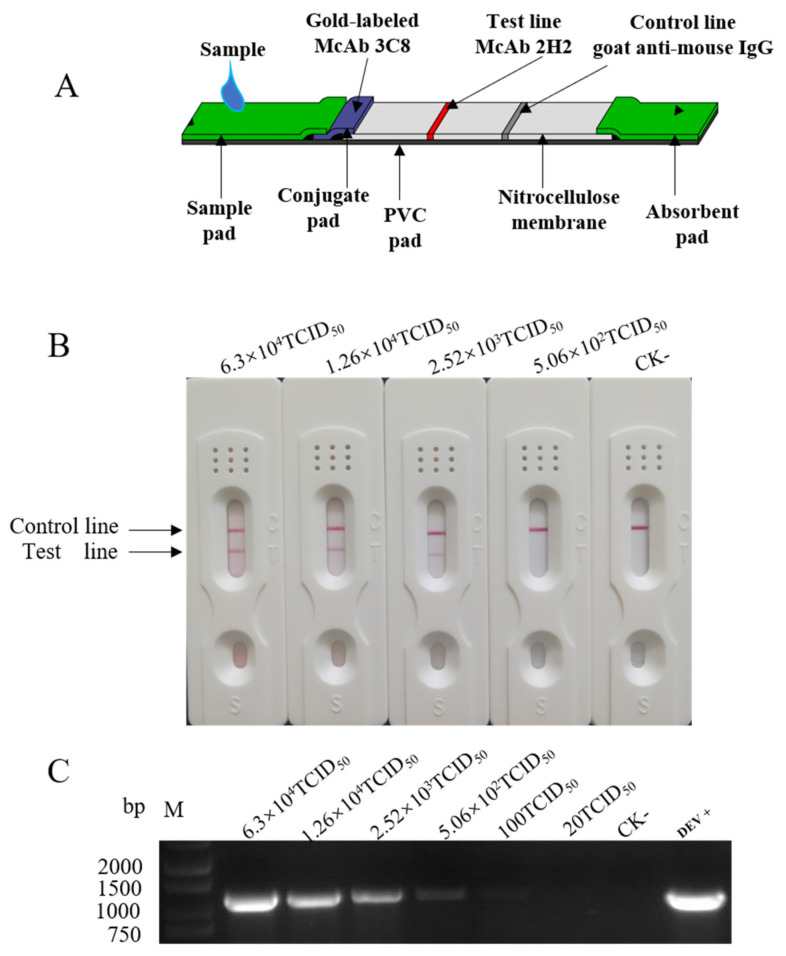
Analytic sensitivity test of the immunochromatographic assay (ICA). (**A**) Schematic diagram of the ICA test. The monoclonal antibody (MAb) 3C8 was conjugated with colloidal gold particles (in blue); the MAb 2H2 was used as a capture antibody in the test line (in red). Goat anti-mouse IgG was used in the control line. The sample pad and absorbent pad were placed with a 1–2 mm overlap on either end of the nitrocellulose (NC) membrane. (**B**) Sensitivity of the ICA. (**C**) Sensitivity of the PCR. A total of 1 × 10^4.5^ TCID_50_/100 μL of the duck enteritis virus (DEV) suspension was serially diluted in five-fold dilutions ranging from 5 × 10^1^–5 × 10^6^. CK-, negative control.

To assemble the ICA strip, as shown in Figure 3A, the MAb 3C8 coupled with colloidal gold was adsorbed on a conjugate pad (72.5 μg/strip) and applied to the base of a plastic-based conjugate pad. With a distance of 9 mm, saturated ammonium sulfate (SAS)-purified ascites MAb 2H2 (92 μg/strip) were immobilized on the nitrocellulose (NC) membrane at the test line and goat anti-mouse IgG (136 μg/mL) was applied 0.5 cm apart as the control line.

To determine the detection sensitivity, serially diluted cell cultured DEV suspensions were examined using the established ICA strips. As shown in Figure 3B, the intensity of the reddish color observed at the test line correlated with the titer of the infectious virus in the cell culture suspension with a detection limit of 2.52 × 10^3^ TCID_50_/mL. In parallel, the PCR assay detected the viral genome at a limit of 1.0 × 10^2^ TCID_50_/mL (Figure 3C), which was 25-fold more sensitive than the ICA. 

To establish the analytical specificity of the ICA, culture suspensions containing viruses commonly encountered in commercially raised duck flocks (duck Tembusu virus, goose parvovirus, duck hepatitis A virus, duck reovirus and avian influenza virus subtypes H5 and H7) and other related herpesviruses (swine pseudorabies virus, Marek’s disease virus and turkey herpesvirus) were examined using the strips to test the diagnostic specificity. No positive signal was observed (Supplementary Materials Figure S1) including Marek’s disease virus and turkey herpesvirus, which also belong to the genus *Mardivirus* alongside the DEV. Furthermore, the virus antigens were clearly detected in the cell cultures infected with six different DEV strains isolated from 2004 to 2014 in China (Supplementary Materials Figure S3). These results indicated a 100% diagnostic specificity for the ICA.

### 2.3. Validation of the ICA Strip Detection with Samples from Experimentally Inoculated Ducks

To evaluate the applicability of the ICA for clinical diagnosis, serial cloacal swabs were collected from live ducks at different time points after experimental infection with the DEV. These samples were tested by ICA and PCR in parallel (Table 1). The DEV antigen in the swabs was first detected by ICA from day three post-infection (p.i.). The overall diagnostic sensitivity of the ICA for the detection of DEV in the cloacal swabs was 87.5–100%, except for one out of the ten swab samples that was positive for DEV genomic DNA by PCR at day two p.i.

**Figure 4:**
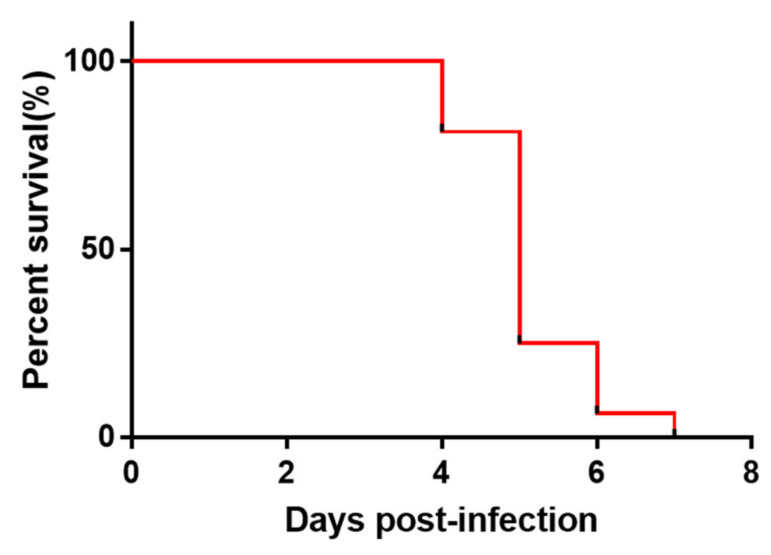
Daily survival percentages of ducklings infected with 5 × 10^3.5^ TCID_50_ of the duck enteritis virus strain with the SD suspension.

Mortality in the DEV infected group started from day 4 p.i. (Figure 4). Duck tissue samples were tested using the ICA strips. As shown in Table 2, most of tissues from the dead ducks were positive by the ICA test, demonstrating a better agreement with the PCR detection. The multiplex strip test results of the tissue homogenates revealed different tissue test sensitivity; the heart and brain had a low test sensitivity that may possibly be related to the nature of the tissues. It is worth noting that livers sometimes have false positive reactions due to non-specific adsorption so we suggest not using the liver for the ICA test on-site.

### 2.4. Stability of the ICA Strip

**Figure 5:**
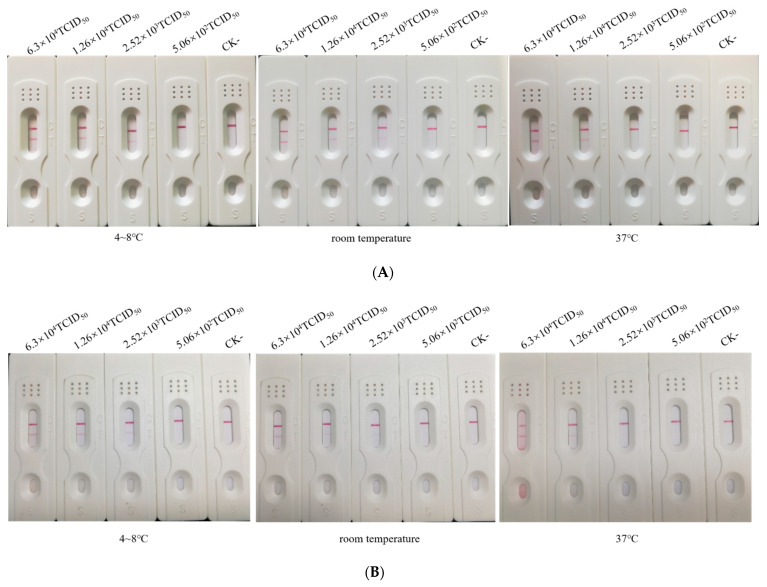
Stability and detection sensitivity of the immunochromatographic assay strips stored for six months (**A**) and three months (**B**) under conditions of 4–8 °C, room temperature and 37 °C.

To evaluate the stability of the ICA strip during storage, the test strips were stored in closed plastic packaging at 4–8 °C, room temperature or 37 °C. The strips retained their initial reactivity and detection limit for cell culture virus suspensions after six months storage at 4–8 °C and room temperature (Figure 5A) whereas the detection limit of strips stored at 37 °C declined to 1.26 × 10^4^ TCID_50_ /mL after three months (Figure 5B). These results indicated that the test strips remained stable at 4–8 °C or at room temperature after six months of storage. 

## 3. Discussion

Herein we have described the development of an ICA strip based on MAbs recognizing the gB and UL47 proteins for the detection of DEV in cloacal swabs and tissue samples. This strip test is portable and easy to use for on-site diagnosis, being evaluated visually without any special equipment. It does not require special training or tools and yields rapid results within 15 min. The results of the ICA demonstrated a high degree of concordance with PCR detection. The specificity and sensitivity of the developed test strip in the current study was comparable with the conventional PCR method (Figure 3B and C). The results from the stability test indicated that the storage of the ICA strip at room temperature for six months did not impact its performance, making this test suitable for use in ambient temperature environments.

As the goal was to detect DVE early following the onset of symptoms, we used pelleted virion particles as an immunizing antigen for antibody preparation to maximize the capture of virus antigens from infected samples. As expected, MAbs recognizing the DEV envelope protein gB and tegument protein UL47 were generated. gB is one of the major glycoproteins in the herpesvirus envelope; it is required for infectivity and functions in the penetration of cells by promoting the fusion of the virion with the plasma membrane [17]. UL47 was found to be the most abundant tegument protein, present at ≥1250 copies per virion [18]. Similarly, UL47 has been identified as a component protein of extracellular virions [19]. Both gB and UL47 proteins are abundant antigens in mature virions and virus infected cell plasma membranes [20]. These highly expressed components are suitable for the recognition of and binding with capture antibodies. The performance characteristics of the ICA were largely dependent on the specificity of the antigen capture antibodies and the DEV titer in the samples. The use of MAbs is advantageous because of their high specificity, sensitivity and uniform affinity for specific antigens. MAbs 3C8 and 2H2 used for the strip assembly recognized the native and recombinant DEV gB and UL47, respectively. It was noted that 3C8 was specific for epitopes of gB from virions and baculovirus-expressed protein (Figure 2B,C), indicating that this epitope was confirmation-dependent. This also explained why this MAb failed to recognize *E.coli*-expressed recombinant gB in this study (data not shown). The observation that both native and baculovirus-expressed gB were recognized as two bands in a western blot analysis suggests that DEV gB is likely cleaved during assembly into virus particles. An examination of the gB peptide sequence revealed a furin cleavage site (^72^RARR^75^), resulting in two bands with relative mobilities estimated at 115 kDa and 105 kDa. This was in accord with the known properties of gB in other herpesviruses [20,21,22].

The aim of the ICA strip development was the detection of the DEV antigen in clinical samples and so virus titer in prospective samples is critical. The DEV replicates primarily in the mucosa of the digestive tract before spreading, resulting in pathological lesions in many different organs [1]. However, the pantropic characteristics of the DEV infection benefited from sample preparation. The test correctly detected positive results in tissue samples prepared from experimentally infected ducks (Supplementary Materials Figure S2). For the two uninfected duck liver samples exhibited by ICA, we excluded the non-specific bind of the MAbs by a double-antibody sandwich ELISA. The possible explanation was that an unknown component in the individual liver sample may have interfered with the reaction. Interestingly, in the ICA detection of *Streptococcus agalactiae* in tilapia, a false positive was observed for uninfected fresh liver samples [23]. Therefore, we suggest that the liver tissue is not used as the only test sample for the ICA diagnosis for the DEV. Under field conditions, the on-site preparation of cloacal swab samples is much easier to perform than that of visceral organs. Using the ICA, the virus antigen was first detected in cloacal swabs from day three p.i. with an 87.5% concordance with conventional PCR detection and false positive results were not found. This finding suggests that the developed strips would be effective in the rapid identification of virus shedding during early symptom onset as the incubation period of most DVEs ranges from 3–7 days [24].

Our study has a few limitations. In particular, the performance of the ICA strips in field outbreaks is as of yet underdetermined and the long-term stability needs to be evaluated. 

In summary, the ICA test developed in this study represents a means for the rapid and inexpensive detection of viral antigens to confirm DEV infection. The test exhibited a comparable sensitivity to that of a conventional PCR while taking less than 15 min to yields results, which would allow a rapid diagnosis and early control of epizootics.

## 4. Materials and Methods

### 4.1. Preparation and Titration of the DEV

The DEV strain SD was isolated from an infected meat-type Pekin duck flock as previously described [25]. The virus was propagated in primary duck embryo fibroblast (DEF) cultures made from 10-day-old Pekin duck embryos by the conventional method. When the cytopathic effect (CPE) reached approximately 75%, the infected cell culture was harvested by three freeze-thaw cycles and inactivated by adding formalin at a final concentration of 0.15% (*v*/*v*) at 37 °C for 16 h. After inactivation, the virus suspension was clarified by centrifugation at 10,000× *g* for 45 min at 4 °C and the clear supernatant was ultracentrifuged at 160,000× *g* for 2.5 h at 4 °C. The pellet was resuspended with sterile PBS in 1/100 of the original volume and stored at −75 °C as the virus antigen.

For the virus titer determination, subconfluent DEF monolayers in 96-well plates were infected with 10-fold serially diluted DEV and incubated at 37 °C. The CPE was recorded daily for seven days. The mean tissue culture infective dose (TCID_50_) was calculated by the Reed and Muench method [26]. 

### 4.2. Detection of the DEV Genome by PCR

For DEV genomic detection, total DNA was extracted from virus infected DEF suspensions or duck tissue samples using a virus DNA kit (Tiangen Co., Ltd, Beijing, China) following the manufacturer’s protocol. The PCR detection primer pair (F: 5′-ATGGGAACGACACGACATAT 3′; R: 5′-TCATACGCTGCCAGTTTTTC-3′) was designed targeting the DEV entire *U_S_7* gene (GenBank: NC_013036.1) with a predicted fragment size of 1089 bp. The thermal cycler was programmed as follows: one cycle at 94 °C for 5 min followed by 30 cycles at 94 °C for 30 s, 55 °C for 30 s and 72 °C for 45 s with a final elongation step at 72 °C for 10 min. Amplification products were run on a 1.5% agarose gel containing ethidium bromide and recorded under UV transillumination.

### 4.3. Production of the Monoclonal Antibody

Mouse immunization and cell fusion were conducted as described previously [27]. Briefly, 8-week-old female BALB/c mice (Beijing Vital River Laboratory Animal Technology Co., Ltd. Beijing, China) were subcutaneously immunized with the concentrated DEV antigen (approximately 1 × 10^5^ TCID_50_/mouse) emulsified with complete Freund’s adjuvant. Two boosters were administrated at two-week intervals with an equal volume of the DEV antigen with an incomplete Freund’s adjuvant. A week after the third immunization, mice were injected intraperitoneally with one dose of the concentrated virus suspension without adjuvant. Three days later, spleen cells collected from immunized mice were fused with SP2/0 cells with polyethylene glycol 2000 (Sigma–Aldrich, St Louis, MO, USA) and cultured in 96-well plates with a layer of macrophage feeder cells collected from the mouse peritoneal cavity. Hybridoma supernatants were first screened using an indirect immunofluorescence assay (IFA) as described in Section 4.4. Positive clones were subcloned by limiting dilution and selected to generate ascitic fluids by the intraperitoneal inoculation of mice. Ascitic fluids were purified by saturated ammonium sulfate (SAS) precipitation [28].

### 4.4. Immunofluorescence Assay

For the IFA, a suspension of DEF was added to 96-well plates at 100 μL per well and the plates were incubated at 37 °C to an 80% confluence. Cells were then infected with the DEV at a multiplicity of infection (MOI) of 0.001 (virus to cell) for 1 h at 37 °C to allow for virus adsorption and the culture was maintained in DMEM with 1% fetal bovine serm (FBS). In parallel, a cell culture plate was maintained as an uninfected control. After 36 h incubation at 37 °C in a CO_2_ incubator, media in the cell wells were removed and the cells were fixed with a pre-chilled acetone/methanol (1:1) mixture for 20 min at room temperature. After washing three times with PBS, a 200 μL blocking buffer (5% skim milk in PBS with 0.1% Tween-20) was added and incubated at 37 °C for 30 min. Wells were then gently washed with PBS and a hybridoma culture supernatant or diluted murine ascitic fluid was added and incubated at 37 °C for 45 min. Wells were washed and DyLight488-conjugated goat anti-mouse IgG (Canlifesci Inc, Co., Ltd., Beijing, China) was added at a dilution of 1:800 followed by 30 min incubation at 37 °C. After three washes, cell nuclei were stained with a blue-fluorescent DNA stain4′,6-diamidino-2-phenylindole (DAPI) (Solarbio, Co., Ltd, Beijing, China) for 10 min at room temperature. Wells were washed again and observed under fluorescence microscopy.

### 4.5. Western Blot and Mass Spectrometric Identification of the DEV Protein

To investigate the protein recognized by the selected MAbs, an ultracentrifugation-concentrated DEV sample was subjected to electrophoresis on a 10% SDS-PAGE in duplicate. After electrophoretic separation, one gel was stained with 0.1% Coomassie Blue R250 to display protein bands and proteins on the other gel were transferred onto a polyvinylidene fluoride (PVDF) membrane. Following overnight blocking with 5% skim milk (in PBS with 0.05% Tween-20), the membrane was probed with a 1:500 diluted SAS-purified ascitic MAb and horseradish peroxidase-conjugated goat anti-mouse IgG at a dilution of 1:5000. The signal was developed with chemiluminescence substrate (ECL reagent; Cwbiotech, Beijing, China).

To identify the proteins recognized by the MAbs, the virus polypeptides were separated by SDS-PAGE under non-reducing conditions and visualized by Coomassie Blue R-250 staining. Gel bands were excised in in-gel digested with trypsin and subjected to N-terminal amino acid sequencing using liquid-chromatography-mass spectrometry (Beijing Protein Innovation Co., Ltd, China).

### 4.6. Expression of the DEV Protein In Vitro

Based the results of the mass spectrometry analysis, the entire DEV UL47 open reading frame (ORF) gene (GenBank: NC_013036.1) was amplified using the primer pair ((F: 5′-ATAAGAATGCGGCCGC**ATGGATAAATCACGAAGACAGCGC**-3′; R: 5′-CGCGGATCC**ATGTAACTCTCTCCGCCCAG**-3′); underlined text shows the Restriction Endonucleases and the UL47 genome sequence is marked in bold) with a predicted fragment size of 2392 bp and cloned into the pMAL-c5X-His vector (New England Biolabs, Inc.). The recombinant UL47 protein was produced in *E. coli BL21(DE3)* following the manufacturer’s protocol. To generate the DEV gB protein, the entire ORF UL27 (GenBank: NC_013036.1) was amplified with the primer pair ((F: 5′-AAAACTGCAGCATCATCATCATCATCAC**ATGTACCGACGGACTATATG-**3′; R: 5′-CCGCTCGAGTCAGTGATGATGATGATGATG**AACTCTGTCTGTGACAAGAA**-3′); the UL27 genome sequence is marked in bold) with a predicted fragment size of 3059 bp. The fragment was inserted into the pFastBac1 plasmid and Sf-9 cells were transfected with the recombinant Bacmid. The baculovirus-expressed protein was produced in Sf-9 cells. 

### 4.7. Double-Antibody Sandwich ELISA and the Conjugation of the MAb with Colloidal Gold

In order to verify the feasibility of if two MAbs could be used to establish an ICA strip, a double-antibody sandwich ELISA was conducted as described previously [29]. Briefly, microtiter plates were coated overnight at 4 °C with 100 μL MAb 2H2 at 2.12 μg/mL each in a PBS buffer. After blocking, 100 μL of a 10-fold diluted tissue suspension were added and incubated for 1 h at 37 °C. After washes with PBS containing 0.05% Tween-20 (PBS-T), 100 μL MAb 3C8-HRP conjugate (diluted 1:5000) were added for 1h at 37 °C. The plate was washed again and developed by incubating with 100 μL/well of a solution of 3,3′,5,5′-Tetramethylbenzidine (TMB, Solarbio, Co., Ltd, Beijing, China). The reaction was stopped by adding 50 μL of 2M H_2_SO_4_ and the absorbance was measured at 450 nm.

A colloidal gold solution was prepared as previously described [30,31]. To prepare the detector reagent, the colloidal gold solution was adjusted to pH 9.0 with potassium carbonate (0.2 M). The MAb 3C8 was coupled to colloidal gold particles as described previously [32]. Briefly, SAS-purified MAb 3C8 (14.5 μg/mL) was added to 1 mL of a 30 nm colloidal gold solution with gentle stirring. After 10 min, 20% bovine serum albumin (BSA) in PBS (*w*/*v*) was added to a final concentration of 0.2% and the solution was stabilized for 10 min. The solution was then centrifuged at 11,100× *g* for 10 min at 4 °C and the soft pellet was resuspended with PBS (0.02 M, pH 7.4) containing 2% sucrose, 0.5% BSA, 0.2% PVP K30, 0.5% Tween-20 and 0.05% ProClin-300. The resuspended solution was sonicated for 3 min and stored at 4 °C.

### 4.8. Determination of Specificity and Sensitivity of the Strip

To evaluate the specificity of the rapid diagnostic strip, the DEV and other viruses commonly reported in domestic waterfowl including duck Tembusu virus(TMUV), goose parvovirus (GPV), duck hepatitis A virus(DHAV-1 and DHAV-3)), duck reovirus (DRV) and avian influenza virus subtypes H5 (H5N1) and H7(H7N9) along with other related herpesviruses including swine pseudorabies virus (PRV), Marek’s disease virus (MDV) and turkey herpesvirus (HVT) were used. To test the reactivity of this ICA to other DEV strains, we used six strains isolated from different regions including DEV 04, DEV BZ, DEV NIU, DEV MA, DEV LIN and DEV SD04. The reactivity of the strip was examined with cell culture supernatants or allantoic fluids containing each virus and the appearance of brown-colored lines in both the control and test windows was judged as a positive reaction. The sensitivity of the strip was assessed using cell cultures infected with serial five-fold dilutions of DEV SD and the detection limit was expressed as log10 TCID_50_.

### 4.9. Duck Infection and Sample Collection

One-day-old Pekin ducklings were purchased from the hatchery of Beijing Golden Star Co., Ltd, Beijing, China. Ducks were raised in two isolators with negative pressure and group 1 (n = 16) were infected subcutaneously with 5 × 10^3.5^ TCID_50_ of DEV SD per duck at 14 days old. Group 2 (n = 10) were inoculated with 0.5 mL sterile PBS as uninfected controls. Cloacal swabs were collected daily until death. Ducks succumbing to disease during the experimental period were autopsied and tissues were collected individually. All samples were stored at −80 °C until use. When tested, tissues were homogenized to 5% (M/V) suspension using sterile PBS (0.01 M, pH = 7.4) including 0.2% Tetronic 1307 (JN Bio, Co., Ltd., Beijing, China). After three rounds of freeze-thaw, the homogenates were centrifuged at 10,000× *g* for 10 min at 4 °C and 100 μL of supernatant were used for the strip test.

### 4.10. Stability of the ICA Strip

To evaluate the stability of the ICA strip during storage, the test strips were stored in closed plastic packaging at 4–8 °C, room temperature or 37 °C up to six months. The sensitivity of the strip was assessed using cell cultures infected with serial five-fold dilutions of DEV SD three times, once a month.

## Figures and Tables

**Table 1:** Test results of the immunochromatographic assay strip and the PCR for duck cloacal swabs.

Days Post-Infectionš(dpi)	Control		Infected		
	PCR	ICA	PCR	ICA	Coincidence (%)
2	0/6	0/6	1/10 *	0/10	93.7
3	0/6	0/6	10/10	8/10	87.5
4	0/6	0/6	10/10	10/10	100
5	0/6	0/6	5/5	5/5	100
6	0/6	0/6	3/3	3/3	100
7	0/6	0/6	1/1	1/1	100
Total	0/36	0/36	30/39	27/39	92.3

* Positive number ducks/total sample number (swabs of infected and control ducks were collected).

**Table 2:** Test results of the immunochromatographic assay strip and the PCR for duck tissue homogenates.

Tissues	Sample Number		Positive Number						Coincidence (%)
			PCR			RICA			
	Control	Infected	Control	Infected	Total *	Control	Infected	Total *	
Heart	5	5	0	5	5/10	0	2	2/10	70
Liver	5	10	0	10	10/15	2	10	12/15	86.7
Spleen	4	10	0	10	10/14	0	10	10/14	100
Lung	5	8	0	8	8/13	0	8	8/13	100
Kidney	4	10	0	10	10/14	0	10	14/14	100
Brain	4	5	0	5	5/9	0	2	2/9	66.7
Bursa	5	10	0	10	10/15	0	10	10/15	100
Intestine	4	10	0	10	10/14	0	10	10/14	100
Total	36	68	68/104			64/104			96.2

* Positive number ducks/total sample number.

## Data Availability

All data presented in this study are available in this manuscript and supplemental materials.

## Supplementary Materials

The following are available online at https://www.mdpi.com/2076-0817/10/3/365/s1, Figure S1: Specificity of the strip. Figure S2: Results from the RICA strip for tissues obtained from experimental samples. Figure S3: Results from the RICA strip for six strains of DEV obtained from different regions. Table 1: Results from the PCR and double-antibody sandwich ELISA for the tissues.
