# Supplementary material for: Development of a Colloidal Gold Immunochromatographic Assay for Duck Enteritis Virus Detection Using Monoclonal Antibodies
Source: Pathogens. 2021 Mar 18;10(3):365. doi: 10.3390/pathogens10030365 (PMC8003186; doi:10.3390/pathogens10030365)
Supplement: Supplementary file 1 [file pathogens-10-00365-s001.pdf]

SUPPLEMENTARY MATERIAL

Development of a colloidal gold immunochromatographic assay for duck enteritis virus detection using monoclonal antibodies

Fengli Liu [1], Yanxin CAO [1], Maokai YAN [1], Mengxu SUN [1], Qingshui ZHANG [1], Jun WANG [2], Guanghua FU[3], Rongchang LIU[3], Yu HUANG[3] and Jingliang SU [1,*]

[1] Key Laboratory of Animal Epidemiology of the Ministry of Agriculture, College of Veterinary Medicine, China Agricultural University, Beijing 100193, China; liufengli0216@163.com
[1] Key Laboratory of Animal Epidemiology of the Ministry of Agriculture, College of Veterinary Medicine, China Agricultural University, Beijing 100193, China; laura12390@163.com
[1] Key Laboratory of Animal Epidemiology of the Ministry of Agriculture, College of Veterinary Medicine, China Agricultural University, Beijing 100193, China; yanmaokai1993@126.com
[1] Key Laboratory of Animal Epidemiology of the Ministry of Agriculture, College of Veterinary Medicine, China Agricultural University, Beijing 100193, 15101065508@163.com
[1] Key Laboratory of Animal Epidemiology of the Ministry of Agriculture, College of Veterinary Medicine, China Agricultural University, Beijing 100193; freedream@cau.edu.cn
[2] Agricultural and Environmental Branch, Jiaxing Vocational and Technical College, Jiaxing, Zhejiang 314036, China; junwang@jxvtc.edu.cn
[3] Institute of Animal Husbandry and Veterinary Medicine, Fujian Academy of Agricultural Sciences, 350013 Fuzhou, China; fuyuan163@163.com
[3] Institute of Animal Husbandry and Veterinary Medicine, Fujian Academy of Agricultural Sciences, 350013 Fuzhou, China; liurongc@foxmail.com
[3] Institute of Animal Husbandry and Veterinary Medicine, Fujian Academy of Agricultural Sciences, 350013 Fuzhou, China; huangyu-815@163.com
* Correspondence: suzhang@cau.edu.cn; Tel.: +86-10-6273-2312

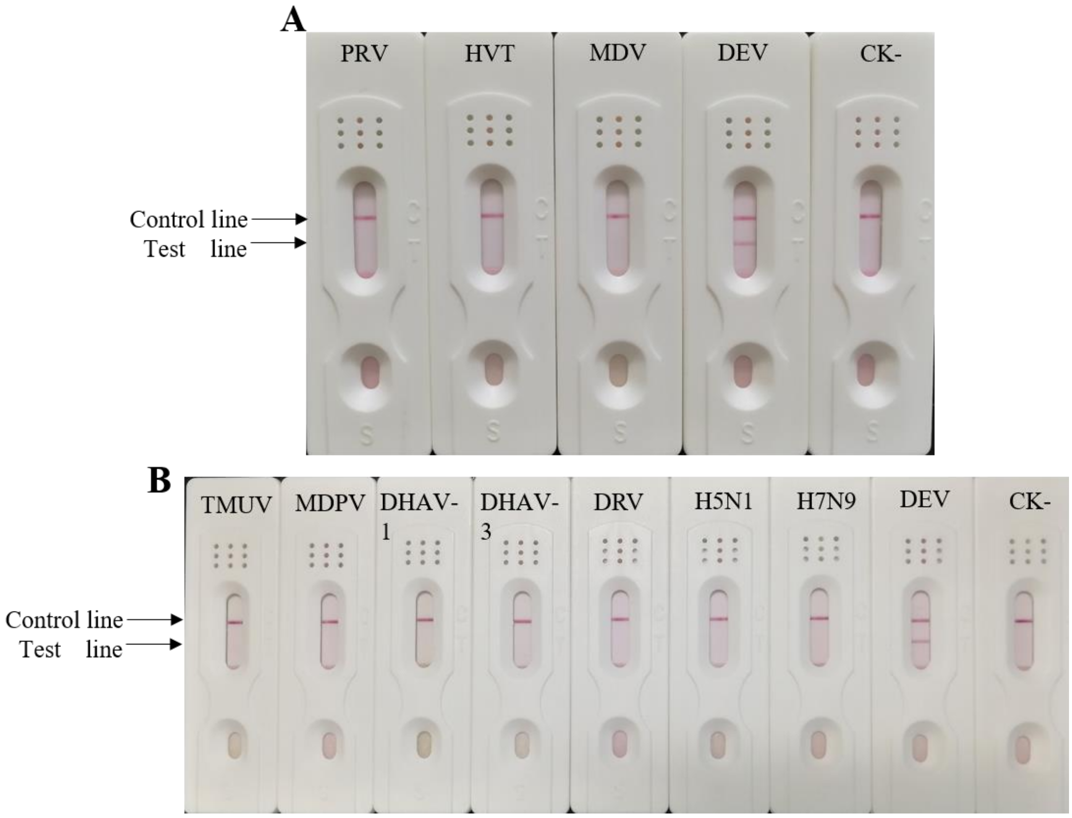

Supplementary Materials Figures S1. Specificity of the strip. (A) The RICA specificity was determined using cell suspensions infected with swine pseudorabies virus (PRV), turkey herpesvirus (HVT), Marek's disease virus (MDV), and DEV, respectively. CK-: Uninoculated cell suspensions were used as negative control. (B) The RICA specificity was determined using viruses commonly reported in commercial duck flocks. including duck Tembusu virus （TMUV）, Muscovy duck parvovirus（MDPV）, duck hepatitis A virus 1（DHAV-1）, duck hepatitis A virus 3（DHAV-3）, duck reovirus （DRV）, avian influenza virus subtypes H5（H5N1）, avian influenza virus subtypes H7 （H7N9）, DEV, CK- Uninoculated cell suspensions were used as negative control.

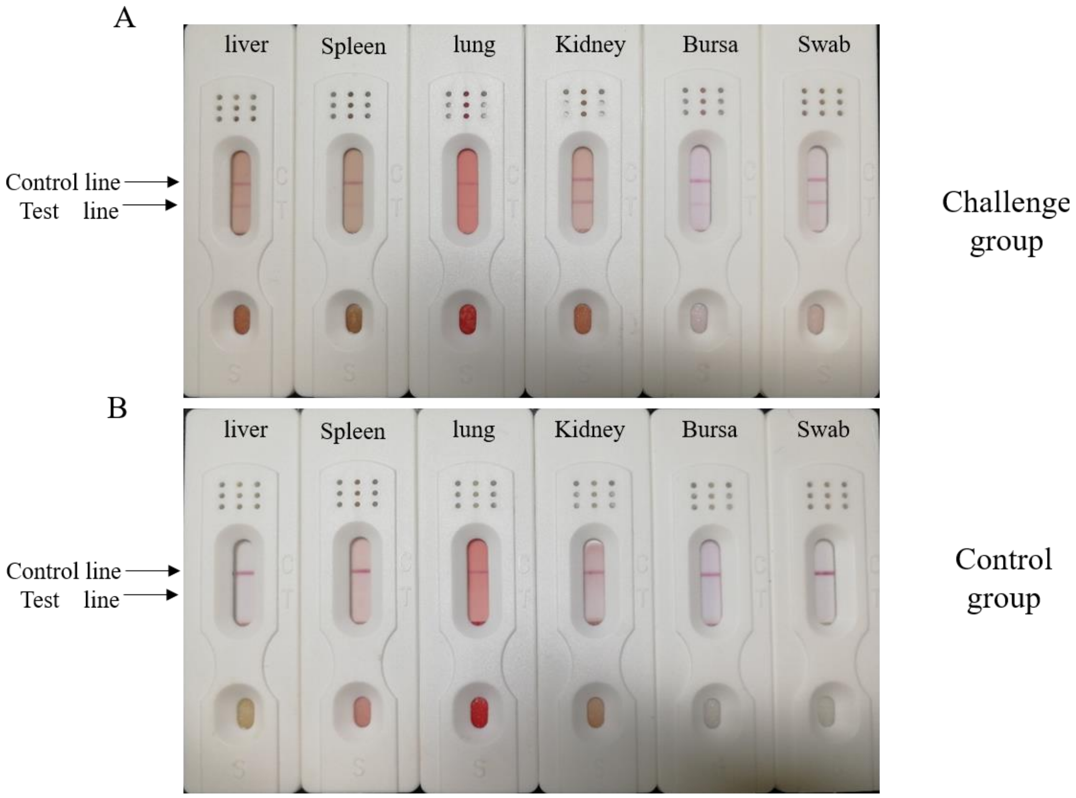

Supplementary Materials Figures S2. Results from the RICA strip for tissues obtained from experimental samples, including liver, spleen, lung, kidney, and bursa. Samples were collected from challenge and control groups for testing. (A) Results of RICA in the challenge group. (B) Result of RICA in the control group.

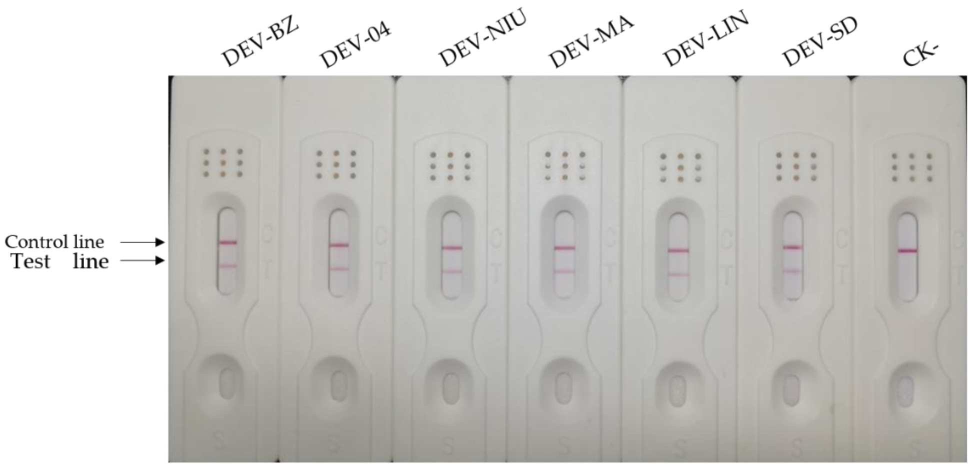

Supplementary Materials Figures S3. Results from the RICA strip for 6 strains DEV obtained from different regions, including DEV-BZ, DEV-04, DEV-NIU, DEV-MA, DEV-LIN, DEV-SD. CK-: control group.

Supplementary Materials Table 1: Results from the PCR and double-antibody sandwich ELISA for the tissues.

tissues	number	PCR		Double-antibody sandiwich ELISA		Coincidence (%)
		+	-	+	-	
liver	103	99	4	99	4	100
kidney	9	5	4	5	4	100
spleen	8	3	5	3	5	100
bursa	5	2	3	2	3	100
Intestine	3	3	0	3	0	100
lung	4	2	2	2	2	100
heart	2	0	2	0	2	100
brain	2	0	2	0	2	100
total	136	114	22	114	22	100
